# A prospective study of urinary pneumococcal antigen detection in healthy Karen mothers with high rates of pneumococcal nasopharyngeal carriage

**DOI:** 10.1186/1471-2334-11-108

**Published:** 2011-04-27

**Authors:** Paul Turner, Claudia Turner, Napaphat Kaewcharernnet, Naw Yi Mon, David Goldblatt, François Nosten

**Affiliations:** 1 Shoklo Malaria Research Unit, Mae Sot, Thailand; 2 Mahidol-Oxford Tropical Medicine Research Unit, Bangkok, Thailand; 3 Centre for Tropical Medicine, University of Oxford, Oxford, UK; 4 Institute of Child Health, University of London, London, UK

## Abstract

**Background:**

Detection of *Streptococcus pneumoniae *C-polysaccharide in urine is a useful rapid diagnostic test for pneumococcal infections in adults. In young children, high rates of false positive results have been documented due to detection of concurrent nasopharyngeal pneumococcal carriage. The relationship between pneumococcal carriage and urinary antigen detection in adults from developing countries with high pneumococcal carriage prevalence has not been well established.

**Methods:**

We nested an evaluation of the BinaxNOW *S. pneumoniae test *within a longitudinal mother-infant pneumococcal carriage study in Karen refugees on the Thailand-Myanmar border. Paired urine and nasopharyngeal swab specimens were collected from 98 asymptomatic women at a routine study follow-up visit. The urine specimens were analyzed with the BinaxNOW test and the nasopharyngeal swabs were semi-quantitatively cultured to identify pneumococcal colonization.

**Results:**

24/98 (25%) women were colonized by *S. pneumoniae *but only three (3%) had a positive BinaxNOW urine test. The sensitivity of the BinaxNOW test for detection of pneumococcal colonization was 4.2% (95% CI: 0.1 - 21.1%) with a specificity of 97.3% (95% CI: 90.6 - 99.7%). Pneumococcal colonization was not associated with having a positive BinaxNOW test (odds ratio 1.6; 95% CI: 0.0 - 12.7; p = 0.7).

**Conclusions:**

Significant numbers of false positive results are unlikely to be encountered when using the BinaxNOW test to diagnose pneumococcal infection in adults from countries with moderate to high rates of pneumococcal colonization.

## Background

*Streptococcus pneumoniae *is a leading bacterial cause of pneumonia globally [1]. Identifying pneumococcus as the etiological agent in pneumonia cases is difficult since unequivocal confirmation requires isolation of the organism from a sterile site. However, only ~10% of pneumonias are bacteremic and few sites are equipped to obtain lung tissue from pneumonia patients [2,3]. Detection of C-polysaccharide in urine (the BinaxNOW *S. pneumoniae *test (Inverness Medical, USA [now marketed by Alere Inc, USA])) has been demonstrated to be a useful rapid confirmatory test for pneumococcal infection in adults, with sensitivity of 74% and specificity of 94% in a recent systematic review [4]. Unfortunately the test has poor specificity in children due to detection of pneumococcal nasopharyngeal colonization: 22 - 67% of healthy children colonized with *S. pneumoniae*, from various countries, have been found to have a positive BinaxNOW test [5-9]. The effect of nasopharyngeal colonization on BinaxNOW test performance in adults has not been widely reported. Studies on the diagnostic utility of the BinaxNOW test in adults are dominated by work from high income countries, where pneumococcal carriage rates are relatively low, e.g. 8% in the UK [10]. A single study has included a formal assessment of the influence of concurrent pneumococcal colonization on the BinaxNOW result: 12% of Spanish HIV-positive patients in the study control group were colonized with *S. pneumoniae *but none had a positive urinary antigen test [11]. Adult pneumococcal carriage rates in lower income countries are often much higher than in developed countries. We have recently demonstrated pneumococcal carriage in 19-27% of young mothers in a Karen refugee population [12]. If this high rate of colonization translates to the presence of antigen in the urine it may limit the diagnostic utility of the BinaxNOW test in certain populations.

In this study we sought to determine whether, in an area with a moderate to high pneumococcal carriage rate in young adults, the BinaxNOW *S. pneumoniae *urinary antigen test detected asymptomatic pneumococcal nasopharyngeal colonization which would limit its utility as a diagnostic test for pneumococcal infection.

## Methods

The evaluation was nested within a longitudinal pneumococcal carriage study conducted in Maela, a densely populated camp for Burmese refugees mainly of Karen ethnicity, 500 km northwest of Bangkok, Thailand [12]. Monthly nasopharyngeal swabs (NPS) were collected from 234 infants and their mothers from birth until 24 months of age. NPS were collected into STGG transport medium (prepared in-house) and processed according to the WHO pneumococcal carriage detection protocol [13].

Between February and April 2010, mothers were asked to provide a urine specimen at a scheduled NPS visit. Women were excluded from participating if they had been treated for pneumonia in the preceding three months or had any symptoms of respiratory infection (fever, earache, coryza, cough, or chest pain) in the preceding six weeks. Urine specimens were collected into sterile containers (Sterilin, UK) and stored at 2-8°C until analysis.

BinaxNOW *S. pneumoniae *urinary antigen testing was undertaken on the day of specimen collection, following the manufacturer's instructions. Nasopharyngeal swabs were frozen at -80°C for up to seven weeks prior to culture. Briefly, the NPS in STGG were fully thawed and 10 μl was cultured on Columbia CNA agar with 5% sheep blood (bioMerieux, France). Plates were incubated overnight at 36°C in 5% CO_2_. *S. pneumoniae *was identified by colonial morphology and optochin susceptibility (zone diameter ≥14 mm) with confirmation by bile solubility when the optochin zone diameter was 7-13 mm. Colonization density was assessed by estimating the number of pneumococcal colonies in each of the four agar plate quadrants: 1+ (growth in quadrant 1 [inoculation area/primary streak] but <10 colonies in quadrant 2), 2+ (>10 colonies in quadrant 2 but <10 colonies in quadrant 3), 3+ (>10 colonies in quadrant 3 but <10 cols in quadrant 4), or 4+ (>10 colonies in quadrant 4). Dense colonization was defined as 2+ or more growth of *S. pneumoniae *on the primary culture plate. Laboratory personnel processing the NPS were unaware of the urinary antigen results.

Data were entered into a Microsoft Access 2003 database (Microsoft, USA) and systematically checked for errors. Statistical analysis was carried out in STATA 10.1 (StataCorp, USA): participant's ages were described by the median and range since non-normally distributed; sensitivity and specificity were calculated using the "diagt" module [14]. Two-tailed p-values of <0.05 and odds ratios with 95% confidence intervals not spanning 1.0 were considered significant.

Written consent was gained from all participants prior to enrollment in the study. Ethical approval was granted by the Ethics Committee of The Faculty of Tropical Medicine, Mahidol University, Thailand (MUTM 2009-306) and the Oxford Tropical Research Ethics Committee, Oxford University, UK (031-06).

## Results

One hundred urine specimens were collected: 98 specimens were accompanied by a NPS and these urine-NPS pairs are considered further. The median participant age was 25 years (range: 15 - 40).

Three of the ninety eight (3%) urine specimens were positive by the BinaxNOW test. From the NPS cultures, 24/98 (25%) of women were colonized by *S. pneumoniae *at the time of urine collection, including 3/24 women (13%) who were densely colonized. The sensitivity of the BinaxNOW test for detection of pneumococcal colonization was 4.2% (95% CI: 0.1 - 21.1%) with a specificity of 97.3% (95% CI: 90.6 - 99.7%) (Table 1). Nasopharyngeal pneumococcal colonization was not associated with a positive BinaxNOW test (odds ratio 1.6; 95% CI: 0.0 - 12.7; p = 0.7).

**Table 1:** BinaxNOW *S.pneumoniae *urinary antigen test and nasopharyngeal swab culture results

		NPS culture		Total

		***S. pneumoniae *** **isolated**	***S. pneumoniae *** **not isolated**	


**BinaxNOW urinary antigen**	**Positive**	1	2	3
	
	**Negative**	23	72	95


	**Total**	24	74	98

Five colonized women were carrying non-typeable (unencapsulated) pneumococci, and therefore would not be expected to develop a positive BinaxNOW test. The remaining 19 colonized women carried typeable pneumococci (Table 2). The single positive BinaxNOW test was from a woman colonized by a serotype 5 pneumococcus. Excluding the five women with NT colonization did not alter the overall results: the sensitivity of the BinaxNOW test for detection of typeable pneumococcal colonization was 5.3% (95% CI: 0.1 - 26%), the specificity was 97.5% (95%CI: 91.2 - 99.7%), and carriage of a typeable pneumococcus was not associated with a positive BinaxNOW test (odds ratio 2.1; 95% CI: 0.0 - 17.5%; p = 0.5).

**Table 2:** Serotypes of carried pneumococci

Serotype	Number of isolates

**3**	1

**5**	2

**6B**	1

**15A**	2

**15B**	1

**17F**	1

**18F**	1

**19F**	3

**19A**	2

**22F**	1

**23F**	1

**28F**	1

**35C**	2

**Non-typeable**	5


**Total**	24

## Discussion

We found that, despite a colonization rate of 25%, only 3% of healthy adult women had a positive pneumococcal urinary antigen test. This 3% (95% CI: 0 - 9%) false positive rate is similar to that found in the systematic review and prospective study of Boulware and colleagues, who reported an overall specificity of 94% (95% CI: 93-95%) for adult pneumonia diagnosis and a 2% false positive rate (1/63) in their non-pneumonia controls (17 HIV positive, and 46 HIV negative) [4]. Interestingly women who had a positive urine antigen result did not have significantly higher odds of being colonized by *S. pneumoniae *than those with a negative test, but the sample size is small. Only one of the women with a positive urinary antigen test had evidence of pneumococcal nasopharyngeal colonization. The other two women gave no history of recent symptoms of, or treatment for, a respiratory infection. It is possible that they may have had a pneumococcal infection several months prior to testing or had recently cleared pneumococcus from the nasopharynx, since detection of pneumococcal urinary antigen is possible at least six months after pneumonia diagnosis in a small number of cases [15].

Compared with the results from studies of young children, it is unclear why, with a carriage rate of 12% in the evaluation of BinaxNOW in Spanish HIV-positive adults, no urine specimens were positive for pneumococcal antigen [11]. There may be differences in how polysaccharides are handled by the immune system in infants compared to adults or there may be issues related to colonization density in adults compared to infants that explain the differences. In our carriage study the overall pneumococcal carriage prevalence was 76% in infants and 24% in mothers. In the infants, dense pneumococcal colonization was observed in a significantly higher proportion of infant culture-positive swabs than mother swabs (47% vs 25%, p < 0.0001) (unpublished data). This may indicate that pneumococcal colonization density plays a role in the high false positive rate for the BinaxNOW test in young children, although in previous studies of children alone have not been able to confirm this [6,9]. A prospective study of urinary antigen detection in infant and adult pneumococcal carriers from the same population may clarify this potential association.

The main limitation of our study was the relatively small sample size: we may have missed a small association between pneumococcal colonization (particularly if this is related to colonization density) and a positive BinaxNOW test as a result of the small numbers of positive urinary antigen tests in our participants. Further studies, either a larger study in our population or in a setting with an even higher prevalence of adult pneumococcal colonization, are warranted to confirm our findings.

## Conclusions

This study provides reassurance that significant numbers of false positive results are unlikely to be encountered when using the BinaxNOW test to diagnose pneumococcal infection in adults from countries with moderate-high rates of pneumococcal colonization. It provides indirect evidence that test performance may be similar in low income countries to that reported from high income country studies. Further studies from areas with even higher adult pneumococcal colonization rates, such as Africa, should be considered. Although these results are encouraging, a considerable reduction in the cost per test would be required to facilitate inclusion of the BinaxNOW test in adult pneumonia diagnostic algorithms in resource-poor settings.

## Competing interests

The authors declare that they have no competing interests.

## Authors' contributions

PT, CT, DG, and FN conceived the study. YM and CT were responsible for specimen and data collection. NK and PT performed the laboratory work. PT did the data analysis and prepared the first draft of the manuscript. All authors reviewed and contributed to revisions of the manuscript.

## Pre-publication history

The pre-publication history for this paper can be accessed here:

http://www.biomedcentral.com/1471-2334/11/108/prepub
